# Artificial Intelligence-Assisted Emergency Department Vertical Patient Flow Optimization

**DOI:** 10.3390/jpm15060219

**Published:** 2025-05-27

**Authors:** Nicole R. Hodgson, Soroush Saghafian, Wayne A. Martini, Arshya Feizi, Agni Orfanoudaki

**Affiliations:** 1 Department of Emergency Medicine, Mayo Clinic Arizona, Phoenix, AZ 85054, USA; 2 Harvard Kennedy School, Harvard University, Cambridge, MA 02138, USA; soroush_saghafian@hks.harvard.edu; 3 Analysis Group, Boston, MA 02199, USA; arshya.feizi@analysisgroup.com; 4 Saïd Business School, University of Oxford, Oxford OX1 1HP, UK; agni.orfanoudaki@sbs.ox.ac.uk

**Keywords:** emergency department, patient flow optimization, vertical processing pathway, machine learning, data-driven patient management, vertical care

## Abstract

**Background/Objectives:** Recent advances in artificial intelligence (AI) and machine learning (ML) enable targeted optimization of emergency department (ED) operations. We examine how reworking an ED’s vertical processing pathway (VPP) using AI- and ML-driven recommendations affected patient throughput. **Methods**: We trained a non-linear ML model using triage data from 49,350 ED encounters to generate a personalized risk score that predicted whether an incoming patient is suitable for vertical processing. This model was integrated into a stochastic patient flow framework using queueing theory to derive an optimized VPP design. The resulting protocol prioritized a vertical assessment for patients with Emergency Severity Index (ESI) scores of 4 and 5, as well as 3 when the chief complaints involved skin, urinary, or eye issues. In periods of ED saturation, our data-driven protocol suggested that any waiting room patient should become VPP eligible. We implemented this protocol during a 13-week prospective trial and evaluated its effect on ED performance using before-and-after data. **Results**: Implementation of the optimized VPP protocol reduced the average ED length of stay (LOS) by 10.75 min (4.15%). Adjusted analyses controlling for potential confounders during the study period estimated a LOS reduction between 7.5 and 11.9 min (2.89% and 4.60%, respectively). No adverse effects were observed in the quality metrics, including 72 h ED revisit or hospitalization rates. **Conclusions:** A personalized, data-driven VPP protocol, enabled by ML predictions, significantly improved the ED throughput while preserving care quality. Unlike standard fast-track systems, this approach adapts to ED saturation and patient acuity. The methodology is customizable to patient populations and ED operational characteristics, supporting personalized patient flow optimization across diverse emergency care settings.

## 1. Introduction

Emergency department (ED) vertical processing pathways (VPPs), in which emergency physicians (EPs) assess and treat patients without assigning them to a traditional ED bed, are increasingly utilized. However, selecting appropriate patients for a VPP remains challenging. Existing studies report varying criteria based on emergency severity index (ESI) levels, but there is minimal literature identifying optimal chief complaints for VPP routing [1]. Ideal patient selection likely depends on institutional ED resources; for example, intravenous line placement may not be possible if patients must return to the waiting room after the initial assessment.

Recent advancements in artificial intelligence (AI) and machine learning (ML) have enabled ED directors to optimize patient flow by devising protocols tailored to their institution’s unique ED characteristics [2,3]. To this end, we hypothesized that a ML model could analyze patient triage data to determine a patient’s suitability for vertical processing.

We previously reported the characteristics of our ED’s VPP, where EPs collaborated with a designated nurse to select patients for assessment while awaiting an assigned ED bed [4]. Eligibility for our VPP was determined at the physician’s discretion, and all waiting room patients were considered potential candidates. Seeking to improve ED throughput and reduce the inefficiencies present in our VPP model, we redesigned our VPP selection criteria using recommendations from a ML model trained to predict whether an ED patient would require an ED bed [5]. We then studied the impact of these resource-neutral changes on ED metrics.

In this article, we demonstrate the application of ML recommendations combined with queueing analyses to improve ED operations, resulting in the creation of a flexible vertical unit unique to the published literature, which personalizes patient care dynamically based on triage data and ED saturation metrics. Our study contributes to the literature by describing a novel use of ML techniques to create a new patient flow option that may appeal to ED medical directors working in resource-constrained environments.

## 2. Materials and Methods

The Mayo Clinic Arizona ED is a tertiary care center located in Phoenix, Arizona, serving approximately 56,000 yearly patients in 56 ED beds. A computerized rotational patient assignment system assigns patients to an EP upon arrival [6]. No triage EP is staffed; instead, the ED relies on EPs to perform chart reviews on waiting patients assigned to them and order the appropriate initial studies to facilitate rapid care. During the pre-trial period, any waiting room patient was eligible for VPP evaluation at the discretion of the assigned EP [4]. After the initial assessment in the VPP, patients would return to the waiting room or a main ED bed once available. Rotating residents participated in approximately 15% of patient encounters in conjunction with an EP. During the study periods, a nurse practitioner or physician’s assistant rotating through the ED conducted an initial evaluation of 9% of patients; however, all patients were ultimately seen and managed by their assigned EP.

To guide the VPP eligibility decisions, we first trained a non-linear binary classification ML model using triage data from 49,350 ED encounters to predict the likelihood that an incoming patient would ultimately require an ED bed [5]. We used retrospectively collected electronic health record data from the Mayo Clinic Arizona ED spanning 7 October 2018 to 31 December 2019. Data from 2020 to 2022 were excluded due to the operational disruptions caused by the COVID-19 pandemic. The primary outcome variable was whether a patient’s care ultimately required an ED bed, defined through a tiered labeling approach. First, patients who were seen in the VPP and discharged without being assigned a bed were labeled as not requiring a bed. To expand the training set, we used clinically guided rules to generate two sets of synthetic labels: (1) patients with an ESI of 3 who were discharged from the ED without imaging or IV therapy and had a total LOS under two hours and (2) patients with an ESI of 4 or 5 who received no IV medications or fluids. All other patients were labeled as requiring a bed. Preprocessing included the removal of duplicate records, exclusion of encounters with missing triage or outcome data, standardization of vital signs, and grouping of chief complaints into clinically meaningful categories based on physician input.

The model outputs a personalized risk score based on each arriving patient’s triage data, including the ESI, chief complaint, age, and vital signs. These predictions were integrated into a stochastic queueing model representing the dynamics of vertical and main ED service processes. Specifically, for the VPP unit, we developed a stylized analytical model using an M/M/1 queue with exponential vacations to reflect the intermittent availability of physicians to staff the VPP, while the main ED was modeled as a typical M/M/1 queue. The model incorporates both patient arrival rates and service times and accounts for the possibility of misclassification by the ML model, namely, routing a patient to the VPP who ultimately requires a bed (type I error) or sending a patient to the main ED who could have been discharged directly via the VPP (type II error). These misclassification trade-offs are embedded in the cost function, which is minimized to determine the optimal routing threshold. Model parameters such as the arrival rates, service times, and VPP reassignment probabilities were estimated from empirical data, and the analytical results were validated using a discrete-event simulation calibrated to our partner ED’s operations. This simulation further compared the VPP design against alternative flow models (e.g., fast-track or physician-in-triage) under varying patient acuity and demand scenarios. The full analytical framework, including queue specifications, parameter estimation, and sensitivity analyses, is detailed in our earlier working paper [5].

**Figure 1:**
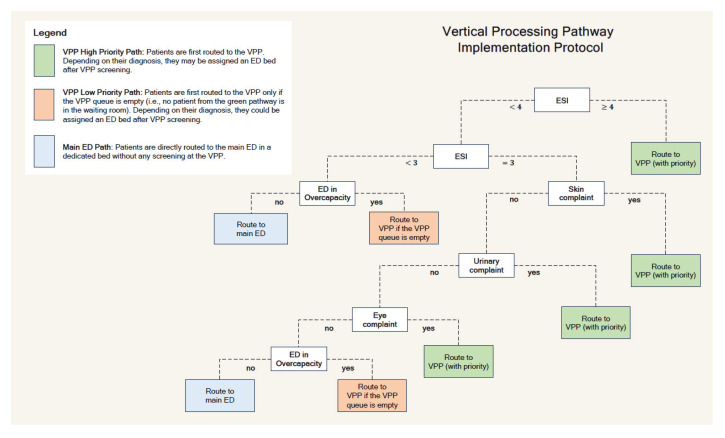
The proposed data-driven VPP implementation protocol [5].

Based on the outputs of this framework, we analytically derived the optimal patient routing threshold that minimizes the time inefficiencies in the system. These thresholds vary dynamically with ED conditions, such as arrival rates and service speed. To operationalize this in practice, we used the analytical model to generate optimal routing labels across a range of empirically calibrated ED states, which were then used, in combination with the patient observations and the non-linear machine learning model predictions, to approximate the optimal patient routing policy. The approximation model resulted in a clinically interpretable and yet personalized decision tree protocol to operationalize VPP assignment in practice. Vertical examination priority was recommended for patients with an emergency severity index (ESI) of 4 or 5, as well as an ESI of 3s, with chief complaints involving skin, urinary, or eye issues. In periods of ED saturation—as defined by the institutional thresholds for patient volume and resource availability—the protocol recommended expanding VPP eligibility to all patients in the waiting room. The final protocol was summarized into a decision tree diagram used by EPs and triage staff during the trial (Figure 1) [5].

To evaluate the protocol, we conducted a 13-week prospective study from 1 February to 30 April 2024. The study was divided into three periods: (1) a pre-intervention period (1 February–5 March), in which the existing ad hoc VPP process continued; (2) an educational period (6 March–26 March), during which clinicians received structured training on the new VPP protocol via virtual sessions and email communication; and (3) a post-intervention period (27 March–30 April), during which the optimized VPP protocol was implemented in clinical practice [5].

To evaluate the impact of the VPP protocol on ED LOS, we estimate multivariable linear regression models on a patient encounter level using the natural logarithm of LOS as the outcome variable [5]. The primary exposure variable is a binary indicator for the post-intervention period. To adjust for potential confounding, we included control covariates from three categories: (1) Patient level: age, ESI, and chief complaint category; (2) Operational: attending physician assignment, disposition status (e.g., discharge, admission), and diagnostic procedures performed (CT scan, ultrasound, X-ray, IV medications); and (3) ED saturation and timing: the number of physicians and nurses on duty at the time of patient arrival, the number of patients currently in treatment or waiting, and the hour of the day grouped into three shifts (6 am–12 pm, 12 pm–6 pm, 6 pm–12 am). These controls are selected to isolate the effect of the VPP intervention from concurrent variations in patient complexity, staffing, and ED congestion. Equivalent analyses were conducted to assess the outcomes of the 72 h ED return rate with or without hospitalization and the potential effect of the protocol on quality of care. Due to anonymization constraints under our IRB protocol, we could not include explicit calendar-based variables (e.g., month or day of the week). However, our models account for seasonal and cyclical variations indirectly through detailed operational controls that capture real-time ED congestion and staffing, which are key mediators of the time-based effects. To assess the robustness of the findings, we ran multiple model specifications, varying the included covariate sets and functional forms (e.g., categorical vs. continuous ESI, physician fixed effects vs. random effects).

## 3. Results

The final machine learning model used for VPP eligibility prediction was a random forest classifier [7], which outperformed alternative algorithms across five bootstrap iterations. On the held-out test set—comprising only “gold-standard” cases with a direct VPP discharge—the model achieved an average AUC of 84.6%, with a low variance across splits, indicating strong generalization. Feature importance analysis [8] revealed that lower ESI scores and abnormal vital signs were associated with an increased likelihood of bed need, while skin, eye, and urinary complaints were the most predictive of safe VPP eligibility.

We summarized the encounter characteristics in the pre-intervention and post-intervention periods in Table 1. With post-intervention, we experienced a significant reduction in ED LOS of 10.75 min (4.15%) with no increase in the 72 h returns or 72 h returns with a hospital admission. Although we did not perform statistical testing on our patient satisfaction scores, our post-intervention “top box score” was 86.0 compared to 85.2 pre-intervention, suggesting no deleterious patient satisfaction effects.

The additional regression analyses, adjusting for clinical, operational, and saturation-related variables, confirmed a statistically significant reduction in ED LOS following the implementation of the optimized VPP protocol. Across multiple model specifications, the estimated adjusted reduction in LOS ranged from 7.5 to 11.9 min (2.89% and 4.60%, respectively), which is consistent with the unadjusted mean difference of 10.75 min that was observed in the raw data. This effect remained robust after controlling for variations in acuity (ESI), patient demographics, physician assignment, diagnostic utilization, ED staffing and saturation, and arrival-time crowding conditions. The protocol’s effect size suggests that the observed improvement in patient throughput was not driven by changes in the patient mix or staffing patterns but was attributable to the introduction of the data-driven VPP streaming approach. Our analyses did not identify any statistically significant effect on the ED return rate outcomes.

Table 2 displays the ESI breakdowns for patients who were initially assessed inVPP pre- and post-intervention. The total number of patients seen through VPP increased during the post-intervention period, representing increased pathway efficiency.

## 4. Discussion

### 4.1. Main Discussion

Despite decades of attention [9], “waiting room medicine” remains a reality for overcrowded EDs nationwide [10]. At the heart of the issue, many contributors to ED crowding are not solvable at the individual ED director level, leading to stopgap measures to try to maintain patient care. Most interventions involve manipulating the ED’s throughput by flexing resources [11] and redistributing personnel, as the physical construction of new care space takes time and may not have the desired effect of decreasing the LOS [12]. However, common patient flow models have inherent pitfalls. Physician-in-triage, for example, requires an additional staffed physician and may lead to rework if the primary treating physician chooses to deviate from the initial triage-based management plan [4]. Similarly, “fast tracks” are best suited to EDs with high numbers of low acuity patients and may not function well in tertiary facilities specializing in complex care; mistriage of patients to a dedicated fast-track practitioner may lead to both rework when reassigned to a non-fast-track physician and to patient safety issues if the mistriage is not identified.

In contrast, specific patient streaming designs [13,14] have demonstrated an advantage in improving an ED’s metrics without additional resource needs or sensitivity to mistriage. The VPP design implemented in our study is a specific patient streaming approach that offers greater flexibility and continuity of care. It allows a single, assigned physician to manage the patient’s care from the initial vertical assessment through to the final disposition, reducing unnecessary transitions and rework. Unlike the “physician-in-triage” approach, it does not require additional physician staffing, and unlike fast-track, it does not rely on strict pre-triage rules to segment patients. Leveraging a non-linear ML model, the VPP is inherently adaptable: during periods of ED saturation, our protocol recommends a dynamic expansion of VPP eligibility based on real-time demands—an advantage not offered by the fast-track or physician-in-triage approaches. Combining ease of implementation with flexibility enables the VPP to function effectively in high-acuity environments where patient needs and ED capacity fluctuate rapidly. Moreover, our rotational patient assignment system, combined with the VPP, allows a patient to remain with the same assigned physician throughout their care episode, eliminating handoffs, reducing redundancy, and allowing physicians to rapidly disposition lower acuity patients while still initiating care for more critical patients during times of ED saturation.

By devising a data-driven VPP protocol that couples the output of ML and stochastic queueing models into interpretable decision-tree-based guidelines, we achieved a significant length of stay reduction while seeing an increased number of patients through the pathway. The protocol’s recommendation to route all ESI 4s and 5s through the VPP is intuitive and sensible, and although our ED treats fewer of these patients than other EDs, most of our lower acuity patients can be served without an ED bed; thus, requiring them to wait for an ED bed creates a significant bottleneck.

The specific ESI-3 chief complaints recommended for routing to the VPP are also reasonable when considering the patient populations. Ocular complaints (outside of visual field cuts, which were characterized as “Neurological Issues” during the analysis due to stroke potential and would typically be an ESI 2 or 1) often do not require imaging but could occupy an ED bed for some time if awaiting an in-person ophthalmologist consultation. Similarly, more complex skin complaints at our facility may require labs or a virtual dermatology consultation, but will typically not require advanced imaging or continuous intravenous access. We were able to manage many urinary issues through the VPP, as well, since we had access to a private area where foley catheters could be placed or pelvic examinations performed; in some EDs, utilizing non-reclining chairs for vertical patients, including this chief complaint category, may not be practical.

While this study was conducted in a single tertiary academic ED and ideal patient selection for vertical processing may differ based on an individual ED’s characteristics, several features of our approach support its broader applicability. First, the protocol relies only on routinely collected triage variables—the ESI, chief complaints, and ED operational status—which are available in most EDs nationwide. Second, the decision tree guidelines were derived based on a site-agnostic stochastic model, matching the theoretical optimal patient routing policy in over 95% of cases, suggesting strong generalizability across the settings. Finally, the implementation did not require dedicated staffing or IT infrastructure changes, further supporting its adoption in resource-constrained environments.

Our implementation of ML-based recommendations to improve VPP patient selection represents a novel use of AI to optimize ED resource allocation. There has been a recent explosion of academic conversation regarding the use of AI to assist EPs with a broad range of tasks. Although many publications present the theoretical benefits of ML protocols based on retrospective data without prospective trials, some recent AI-based inventions are now utilized in cutting-edge EDs. Recognizing the deleterious impacts of the documentation burden on EPs [15], significant investments have focused on tools to summarize portions of the electronic medical record or ambient AI devices to document clinical encounters [16]. Recent publications describe the creation of ML-based predictors of ED patient deterioration that may ultimately improve patient safety; however, most of these systems have yet to be applied in clinical settings [17,18,19].

AI-based patient flow interventions have been somewhat limited in scope, mainly focused on triage. In conventional ED triage, patient prioritization is based on a combination of ESI and triage nurse gestalts. AI-based triage has the potential to improve patient selection through the awareness of real-time ED resource utilization and dynamic patient flow. It has also been shown to limit biases inherent in a human-based triage model [20]. Some current AI-based triage systems, such as KATE [21], serve to assist triage nurses in assigning a more accurate ESI score, while others seek to replace the ESI entirely with a program running concurrently with the medical record. However, many models remain theoretical and have not been trialed in clinical practice [22,23]. Most interventions have focused on accurately predicting resource utilization during triage for a particular patient and have not examined dedicated streaming pathways based on the AI-assigned score.

Our study, one of the first in the literature to showcase the actual clinical implementation of ML-based ED flow recommendations, displays the clinical practice benefits of combining recommendations from advanced analytics techniques into real-world patient flow protocols. We present our results as a potential option for other EDs struggling with overcrowding, enabling EPs to personalize care pathways for presenting patients.

### 4.2. Limitations

This study was conducted under real-world clinical conditions and reflects the challenges inherent in implementing protocol-based changes in a high-volume ED. As such, adherence to the optimized VPP protocol was imperfect. Despite instructions to preferentially route skin, urinary, and eye ESI-3 patients through VPP, the triage nurses had greater success routing ESI 4s, 5s, and non-protocol-eligible ESI 3s (presumably during periods of ED saturation). Only 8.64% of skin, urinary, and eye ESI 3s were routed through the VPP post-intervention; although the ML algorithm suggests that increasing this percentage would further benefit the LOS, the actual application of this increase may have unintended effects. Importantly, the protocol relied on manual physician and nurse interpretations rather than automated integration within the electronic medical record. Embedding real-time VPP eligibility recommendations into the triage interface may improve future adherence but would require dedicated IT infrastructure and development timelines that were not feasible within the scope of this study. Finally, like many other before-and-after studies, the Hawthorne effect might have influenced our results. Despite these, our various checks indicated systematic improvements post-implementation, suggesting a useful patient flow redesign approach that could be implemented across a variety of EDs.

## 5. Conclusions

Using a data-driven quantitative model to personalize the selection of patients seen through a vertical pathway allowed us to treat an increased number of ED patients without the use of an ED bed, reserving these valuable resources for more critical patients and improving the LOS. Beyond demonstrating operational improvements, our study introduces a new, formalized paradigm for VPPs—one that integrates machine learning and stochastic modeling to generate personalized, context-aware streaming decisions. This framework offers a generalizable, scalable, and adaptable approach for EDs seeking to optimize patient flow under resource constraints.

## Figures and Tables

**Table 1:** Encountered characteristics before and after the intervention [5].

	Pre-Intervention (N = 5522)	Post-Intervention (N = 5493)	*p*-Value
**Outcomes**			
**ED Length of Stay** (Minutes [SD])	258.74 (121.88)	247.99 (115.44)	*p* < 0.001
**Admission Status** Discharged (%)	67.08%	66.96%	*p* > 0.05
**ED return within 72 hrs**	3.89%	3.84%	*p* > 0.05
**ED return within 72 hrs with admit**	2.37%	2.17%	*p* > 0.05
**Patient Characteristics**			
**Sex**	53.48%	54.31%	*p* > 0.05
Female (%)			
**Age**	58.92 (20.98)	58.55 (20.92)	*p* > 0.05
Mean Years (SD)			
**Race**	88.52%	88.15%	*p* > 0.05
White (%)			
**ESI** (Mean [SD])	2.88 (0.66)	2.90 (0.64)	*p* > 0.05
**Procedures Administered**			
IV (%)	64.98%	65.83%	*p* > 0.05
CT with IV contrast	24.85%	24.63%	*p* > 0.05
CT without IV contrast	19.76%	19.83%	*p* > 0.05
X-ray	45.60%	44.13%	*p* > 0.05
Ultrasound	11.81%	12.53%	*p* > 0.05

SD = standard deviation; hrs = hours.

**Table 2:** ESI breakdowns for VPP patients pre- and post-intervention.

ESI	Pre-Intervention	Post-Intervention	Statistical Test	*p*-Value
1	0 (0.0%)	0 (0.0%)	Fisher	*p* > 0.05
2	4 (0.28%)	7 (0.53%)	Fisher	*p* > 0.05
3—Excluding Skin/Urinary/Eye	47 (1.55%)	163 (5.26%)	χ^2^	*p* < 0.001
3—Skin/Urinary/Eye	3 (1.23%)	21 (8.64%)	Fisher	*p* < 0.001
4	139 (18.08%)	469 (59.97%)	χ^2^	*p* < 0.001
5	7 (20.0%)	19 (82.61%)	Fisher	*p* < 0.001

% represents the percentage of all patients of that ESI during the study period seen in VPP. We used Pearson’s chi-square test to compare the pre- and post-intervention VPP routing proportions for each ESI group, applying Fisher’s exact test instead for any subpopulation of a size less than 10.

## Data Availability

The original contributions presented in this study are included in the article. Further inquiries can be directed to the corresponding author.

## Abbreviations

The following abbreviations are used in this manuscript:

AI	Artificial Intelligence
ML	Machine Learning
ED	Emergency Department
VPP	Vertical Processing Pathway
ESI	Emergency Severity Index
LOS	Length of Stay
EP	Emergency Physician
IV	Intravenous
IRB	Institutional Review Board
AUC	Area Under the Curve
SD	Standard Deviation
hrs	Hours
IT	Information Technology
